# Characteristics of Dyslipidemia in Primary Nephrotic Syndromes

**DOI:** 10.7759/cureus.84077

**Published:** 2025-05-14

**Authors:** Muhannad Alqudsi, Sulaiman Alhumaid, Ali S Almagooshi, Ahmad M Samman, Ali M Alqaraishi

**Affiliations:** 1 Nephrology, King Abdulaziz Medical City Riyadh, Riyadh, SAU; 2 College of Medicine, King Saud Bin Abdulaziz University for Health Sciences College of Medicine, Riyadh, SAU

**Keywords:** dyslipidemia, focal segmental glomerulosclerosis (fsgs), minimal change disease (mcd), primary membranous nephropathy, statin use

## Abstract

Background

Although it is not a criterion for diagnosis, dyslipidemia is frequently found in nephrotic syndrome (NS). Cholesterol, triglyceride, and low-density lipoprotein (LDL) are usually elevated in NS, and high-density lipoprotein (HDL) can be normal or minimally decreased. Dyslipidemia in NS has been studied in isolation of the underlying glomerulopathy, and the comparison of lipid values between membranous nephropathy (MN), minimal change disease (MCD), and focal segmental glomerulosclerosis (FSGS), is not well recognized.

Methods

Retrospective chart review of patients with NS from 2010 to 2022. Patients with primary MN, MCD, and primary FSGS were included. Lipid profile was reported at the time of NS diagnosis and 12 months later. We compared lipid values between three primary NS using Kruskal-Wallis and Mann-Whitney U tests.

Results

There were 409 patients diagnosed with NS. 284 patients were excluded due to insufficient data or a diagnosis of secondary NS. One hundred and twenty-five patients with FSGS, MN, or MCD were included: FSGS (52, 41%), MCD (31, 25%), and MN (42, 34%). The average age was 32 years, with 55 females (44%), and 79 received statins (56%). After adjustment for serum albumin and proteinuria, initial cholesterol and triglyceride levels were similar in the three NS groups (P>0.05). Low-density lipid (LDL) was 216 mg/dL, 201 mg/dL, and 178 mg/dL in FSGS, MCD, and MN, respectively; the difference was only in FSGS vs MN group (p=0.04). Initial HDL was 58 mg/dL, 77 mg/dL, and 50 mg/dL in FSGS, MCD, and MN respectively (p=<0.001), differences were in MCD vs FSGS, and MCD vs MN groups (p=0.001, and p=<0.001 respectively). After 12 months of follow-up, lipid values were similar in the three NS groups regardless of statin use.

Conclusion

After adjustment for primary NS severity, cholesterol and triglyceride values were insignificantly different at the presentation of MN, MCD, and FSGS. HDL was significantly higher in MCD compared to MN and FSGS, and LDL was significantly higher in FSGS compared to MN. At the 12-month follow-up, statin use did not change lipid values.

## Introduction

Regardless of the underlying culprit kidney disease, nephrotic syndrome (NS) is universally defined as a triad of heavy proteinuria, hypoalbuminemia, and edema. Adults with NS have high rates of end-stage kidney disease and cardiovascular-associated death [1]. Dyslipidemia is commonly present in NS; nevertheless, it is not considered a diagnostic criterion and is widely overlooked or over-treated [2,3]. Despite being the most common cause of secondary NS, diabetic nephropathy (DN) can still present frequently without NS features [4], on the other hand, patients diagnosed with primary membranous nephropathy (MN), minimal change disease (MCD), or primary focal segmental glomerulosclerosis (FSGS) almost always express features of NS, which is referred to as primary NS to distinguish it from secondary NS caused by other etiologies such as but not limited to, DN, lupus nephritis, amyloidosis, etc. [5].

Not only is NS common in the nephrology field, but it also adds additional mortality and morbidity risk that is proportionally amplified by the severity of the syndrome [1,6]. Dyslipidemia is almost always encountered in primary NS, and it acts as an independent risk factor for cardiovascular morbidity, yet its effect on renal function and optimal treatment approach remains an uncertain area [3].

Recently, there has been a better understanding of NS mechanisms, and more than one hypothesis has been postulated. Additionally, the pathogenesis of dyslipidemia in NS has been better described. Nevertheless, data are very scarce when it comes to comparing the mechanisms of dyslipidemia and NS between different primary NS.

Although the diagnosis of primary NS-related glomerulopathy is usually straightforward, it can be cumbersome when tissue from a kidney biopsy is suboptimal or difficult to obtain. The recently discovered serological and histological markers can aid in diagnosis, but are typically useful in MN with variable sensitivity, specificity, and availability. In dyslipidemia of primary NS, variations of low-density lipoprotein (LDL), high-density lipoprotein (HDL), and triglyceride exist [7], the statistical significance and medical and diagnostic importance of such variations are rarely reported in the literature if any, such deep dissection of lipid variability can open the door for better understanding of NS mechanisms and aid further as a diagnostic and prognostic tool for patients suffering from primary NS. This article was previously presented as an abstract poster at the 2024 ASN meeting on October 24, 2024.

## Materials and methods

In this single-center retrospective cohort study, we identified patients diagnosed with NS due to primary MN, MCD, and primary FSGS, and followed at King Abdulaziz Medical Center between the years 2010 and 2022. We included patients who were at least 18 years old at the time of diagnosis. Patients diagnosed with NS in childhood were included only if they experienced a relapse during adulthood after being in complete remission. We excluded patients with secondary forms of MCD, MN, or FSGS, and those with pre-existing dyslipidemia. Patients with DM were reported along with their average hemoglobin A1C to exclude patients with an average of > 8 percent. NS diagnosis was confirmed by reviewing the diagnostic criteria of albumin < 3.5 mg/dL, urine protein-creatinine ratio (UPCR) > 3.5 g/g, and evidence of edema. Diagnosis of primary MN, MCD, or primary FSGS was confirmed by the officially reported kidney biopsy in the electronic medical record (EMR), or by the physicians’ documentations of reviewing kidney biopsy done outside our facility. LDL, high-density lipoprotein (HDL), triglyceride, cholesterol, serum albumin and creatinine, and UPCR were reported at the time of NS diagnosis/recurrence and 12 months of follow-up.

The statistical analysis was performed using SPSS software, version 27 (IBM Corp., Armonk, NY). Continuous variables were presented as median and interquartile range (IQR) due to their non-normal distribution. Categorical variables were presented as frequencies and percentages. The Mann-Whitney U test was used to compare continuous variables between two independent groups. The Chi-squared test or Fisher's exact test was used to compare categorical variables where appropriate. The Kruskal-Wallis test was used to compare differences between three or more groups. Significant values were adjusted by the Bonferroni correction for multiple tests. The Wilcoxon signed-rank test was used to compare paired continuous variables. We conducted Spearman correlation to study the association between baseline laboratory tests. Generalized linear analysis was utilized for multivariable analysis to determine the association between study variables and baseline cholesterol. All significant variables in the univariable analysis were included in the multivariable model. A two-sided p-value < 0.05 was considered statistically significant for all tests.

## Results

Between 2010 and 2022, 409 patients were diagnosed with NS. After applying the inclusion and exclusion criteria, 125 patients were included for data analysis. The mean age of the entire population was 36 years. Patients were predominantly male (n=70, 56%), and all were of Arab descent (Saudi). Etiology of NS was primary FSGS in 52 (41.6%), MCD in 31 (24.8%), and MN in 42 (33.6%). Patients who had DM were 25 (20%), all of them had an average hemoglobin A1C of ≤ 8.0. Baseline characteristics of the population based on Primary NS etiology are shown in Table 1.

**Table 1:** Patient characteristics, overall and by primary nephrotic syndrome category *At time of diagnosis or recurrence. DM = Diabetes Mellitus, FSGS = Focal segmental glomerulosclerosis, HDL = High-density Lipoprotein, IQR = Interquartile range, LDL = Low-density Lipoprotein, MCD = Minimal change disease, MN = Membranous nephropathy, UPCR = urine protein/creatinine ratio (g/g).

Characteristic	Overall	FSGS	MCD	MN	P-value
Number (%)	125	52 (41.6%)	31 (24.8%)	42 (33.6%)	
Age median (IQR)	32	30	28	37	0.055
Gender n (%)					
Female	55 (44%)	27 (51.9%)	13 (41.9%)	15 (35.7%)	0.2
Male	70 (56%)	25 (48.1%)	18 (58.1%)	27 (64.3%)	
Race n (%)					
Arab	125 (100%)				
DM n (%)	25 (20%)	14 (26.9%)	2 (6.5%)	9 (21.4%)	0.07
Treatment n (%)					
Steroids	77 (61.6%)	35 (67.3%)	22 (71%)	20 (47.6%)	0.07
Rituximab	25 (20%)	3 (5.8%)	12 (38.7%)	10 (23.8%)	0.001
Other immunosuppression	60 (48%)	24 (46.2%)	13 (41.9%)	23 (54.8%)	0.5
Statin	79 (56%)	31 (59.6%)	14 (45.2%)	25 (59.5%)	0.3
Baseline lipid levels* median (IQR)					
Cholesterol	278.4	290	309	239.7	0.007
LDL	170	201	162.4	154.6	0.02
HDL	54.1	54.1	69.6	50.2	<0.001
Triglyceride	150.5	159.4	141.7	159.4	0.4
Serum creatinine	0.7	0.7	0.7	0.9	0.3
Serum Albumin	2.3	2.4	2.0	2.5	0.04
UPCR	7.3	7.7	6.6	6.9	0.6

At baseline, cholesterol, LDL, HDL, and serum albumin values were significantly different between primary NS, p-values were (0.007, 0.02, < 0.001, and 0.04, respectively). On the other hand, triglyceride, creatinine, and UPCR were the same, p-values were (0.4, 0.37, and 0.68, respectively). Considering serum albumin and UPCR as markers of NS severity, overall correlation with lipid dysregulation was found to be highest between albumin and cholesterol, and lowest between albumin and HDL (correlation coefficient -0.543 and -0.056, respectively) (Table 2).

**Table 2:** Correlations between baseline lipid levels and primary nephrotic syndrome severity Represented by baseline serum albumin and proteinuria. HDL = High-density lipoprotein, LDL = Low-density lipoprotein, r = Correlation coefficient, UPCR = Urine protein/creatinine ratio (g/g).

	HDL	Cholesterol	Triglyceride	LDL
Serum albumin (r)	-0.056	-0.543	-0.075	-0.543
UPCR (r)	-0.116	0.174	0.203	0.172

To adjust for NS severity, we conducted generalized linear analysis with a Gamma distribution and a log link function to adjust for UPCR and albumin (Table 3).

**Table 3:** Baseline lipid values by primary nephrotic syndrome category adjusted for nephrotic syndrome severity Represented by serum albumin and proteinuria. ^a^Difference is between FSGS vs MN and MCD vs MN (P-values 0.04, 0.008, respectively); ^b^Difference is between FSGS vs MCD, and MCD vs MN (P-values 0.002, and < 0.001, respectively). ^c^Difference is between FSGS vs MCD, and MCD vs MN (P-values 0.001, and <0.001, respectively). ^d^Difference is between FSGS vs MN, and MCD vs MN (P-values 0.01 and 0.04, respectively). ^e^Difference is between FSGS vs MN (P-value 0.04). FSGS = Focal segmental glomerulosclerosis, HDL = High-density lipoprotein, IQR = Interquartile range, LDL = Low-density lipoprotein, MCD = Minimal change disease, MN = Membranous nephropathy, UPCR = Urine protein/creatinine ratio (g/g).

	FSGS	MCD	MN	P-value
Cholesterol median (IQR)				
Adjusted for UPCR^a^	313.2	340.3	266.8	0.005
Adjusted for albumin	309.3	324.8	274.5	0.08
HDL median (IQR)				
Adjusted for UPCR^b^	58	73.4	50.2	<0.001
Adjusted for albumin^c^	58	77.3	50.2	<0.001
LDL median (IQR)				
Adjusted for UPCR^d^	224.2	220.4	170.1	0.006
Adjusted for albumin^e^	216.5	201	177.8	0.04
Triglyceride median (IQR)				
Adjusted for UPCR	186	168.2	203.7	0.4
Adjusted for albumin	186	168.2	203.7	0.2

P-values are corrected for multiple comparisons by sequential Bonferroni. We found no difference in cholesterol values between primary NS after adjusting for serum albumin levels, p-value was 0.08, while a significant difference was present in LDL and HDL, p values were 0.04, and < 0.001, respectively. LDL was only significantly different between FSGS and MN, with mean values of 216.5 mg/dL and 177.8 mg/dL, respectively (p-value was 0.04). HDL was significantly lower in FSGS and MN (mean values were 58 mg/dL and 50.2 mg/dL, respectively) compared to MCD (mean value was 77.3 mg/dL), p-value was 0.001, yet it was similar in FSGS and MN, p-value was > 0.05. After 12 months of follow-up, remission rates in FSGS, MN, and MCD were 40 (77%), 21 (67%), and 35 (84%), respectively (p-value was 0.13). Despite this similarity, HDL and triglycerides were significantly different in MCD compared to MN and FSGS, but similar in MN and FSGS. Cholesterol and LDL values were similar in all primary NS (Table 4).

**Table 4:** Lipid values after 12-month follow up by primary nephrotic syndrome category *Difference is between MCD vs MN, and MCD vs FSGS (p-values 0.002 and 0.009, respectively). **Difference is between MCD vs MN, and MCD vs FSGS (p-values 0.04 and 0.02, respectively). FSGS = Focal segmental glomerulosclerosis, HDL = High-density lipoprotein, IQR = Interquartile range, LDL = Low-density lipoprotein, MCD = Minimal change disease, MN = Membranous nephropathy.

	FSGS	MCD	MN	P-value
Cholesterol median (IQR)	181.7	174	185.6	0.6
LDL median (IQR)	116	100.5	100.5	0.3
HDL median (IQR)*	46.4	58	46.4	0.001
Triglyceride median (IQR)**	124	88.5	106.3	0.01

Changes in cholesterol and HDL values over 12 months were similar in statin users vs non-statin users with p-values of 0.93 and 0.67, respectively, and that was regardless of the underlying primary NS.

## Discussion

In this single-center retrospective study, we found some differences in dyslipidemia characteristics between primary NS despite NS being presumed to be the mutual underlying etiology. We also found that lipid dysregulation correlates weakly with the degree of UPCR, yet correlation with serum albumin was more prominent. After adjusting for NS severity, LDL was noted to be significantly higher in FSGS compared to MN, and HDL was significantly lower in FSGS and MN compared to MCD. After 12 months of follow-up, MCD was found to have significantly higher HDL and lower triglyceride. Statins intake did not change the eventual cholesterol, or HDL levels.

Although the pathogeneses of the three common primary NS are different, the microscopic kidney finding of extensive podocytopathy is a hallmark that results in severe proteinuria as part of NS, a condition marked by significant proteinuria, hypoalbuminemia, hyperlipidemia, and edema [8]. The two theories that tried to explain the consequences of NS had their explanations in isolation from the underlying glomerulopathy. The underfill theory states that hypoalbuminemia from massive protein loss reduces plasma oncotic pressure, shifts fluid from the intracellular compartment to the interstitium and causes edema. This decrease of circulatory volume leads to renal hypoperfusion and activation of the renin-angiotensin-aldosterone system (RAAS), which results in sodium and water retention. In contrast, the overfill theory proposes that hypoalbuminemia alone is insufficient to be the culprit, instead primary renal sodium and water retention, and expanded intravascular volume are the key factors driven by overactive epithelial sodium channels (ENaC) in cortical collecting ducts either by increased sodium-potassium adenosine triphosphatase (Na+ K+ ATPase) activity or directly by massive proteinuria [9-12].

Dyslipidemia is a common complication of NS that can result from hypoalbuminemia, which triggers compensatory over-synthesis of hepatic lipoproteins and increased levels of triglycerides and cholesterol. Anther postulated mechanism is the decrease of lipoprotein clearance due to impaired lipoprotein lipase enzyme activity or due to the downregulating of hepatic LDL receptors. HDL values are commonly within or below normal in NS. However, the HDL to total cholesterol ratio is reduced and maturation of cholesterol ester-rich HDL is impaired. Near normal HDL values in NS are not fully understood but can be a result of low serum albumin that is necessary for free cholesterol transfer from peripheral tissues to HDL [3,13]. The minimal change of HDL levels in NS compared to other lipids is well presented in our data by reporting the correlation between serum albumin and HDL, which is the weakest.

All postulations regarding the NS mechanism itself or its associated dyslipidemia do not take into consideration the original underlying pathogenesis when explaining the aforementioned volume and lipid presentations. Our findings show statistically different lipid values among primary NS at the time of diagnosis and as a trajectory, which might strongly suggest different mechanisms of dyslipidemia and possibly of NS depending on the underlying glomerulopathy. In MN, recent studies explored several circulating antibodies believed to be targeting glomerular podocytes, whereas in FSGS and MCD, the attribution to specific circulating agents is less clear. In MCD there is a subtype of patients believed to have autoantibodies targeting nephrin of the slit diaphragm in the glomerular basement membrane, yet the majority of patients have other circulating factors due to T cell and B cell dysfunction, on the other hand patients with FSGS have not shown any specific circulating factors that podocytopathy can be attributed to [14-17]. While the immune system is dysregulated in primary NS, it is unknown whether previous different pathogeneses play roles in certain manifestations of NS and dyslipidemia presentation. For instance, it is possible that higher levels of LDL in FSGS compared to MN could be due to increased circulating factors from activated T-cells, since these factors were found to be contributing to increased hepatic lipoprotein production and activated T-cells can alter the expression of cellular cholesterol transporters [18,19]. Similarly, the higher levels of HDL in MCD compared to FSGS and MN could be related to a less inflammatory process in MCD, which might be associated with less immune circulating factors that are originally responsible for decreased HDL levels [3,20,21].

While patients with hyperlipidemia are considered at high risk for cardiovascular morbidity, the presence of nephrotic syndrome further magnifies this risk [22,23], hence, it is intuitive to treat hyperlipidemia as this has been shown to decrease cardiovascular mortality [24]. Furthermore, evidence exists for hypercholesterolemia, low HDL, and hypertriglyceridemia to be associated with increased proteinuria and likely to worsen CKD progression through the “ectopic lipid accumulation” phenomenon, where certain levels of hyperlipidemia exceed the body’s ability to store fat in adipose tissue, as a result extra lipids spill over into non-adipose tissues including mesangial cells, podocytes, and proximal tubules in kidneys [25-28]. What also supports the previous notion is the improvement of proteinuria and glomerulosclerosis after treating hyperlipidemia, shown in different clinical trials on humans and animals [29,30].

Statins have been widely used in treating dyslipidemia due to compelling data from different studies showing not only a reduction in cardiovascular events in non-NS patients [31], but also improvement in kidney function and proteinuria especially after long term use [32]. Nevertheless, the data in the NS population are inconsistent, while early studies showed the good effect of statin in cholesterol reduction [33], later meta-analysis found this efficacy is limited [34,35]. In real-life practice, many healthcare providers tend to treat dyslipidemia in NS, yet it is wise to weigh the risks vs benefits of such treatment. Using statins as a first line treatment for dyslipidemia is not always benign as it is thought to be. Although related rhabdomyolysis is uncommon [36], other side effects such as acute interstitial nephritis have been reported [37]. In a large study from the UK, statin use was associated with significantly increased risk of acute kidney injury (AKI) within the first year of starting the medication, which was dose dependent and was seen across all statins, except rosuvastatin [38]. In another large study, rosuvastatin was found to predispose patients to proteinuria and hematuria compared to atorvastatin in a dose-dependent manner, especially in patients with estimated glomerular filtration rate (eGFR) of less than 30 ml/min per 1.73 m^2^ [39]. Our data showed no difference in cholesterol values after twelve months of follow-up in primary NS patients regardless of statin use. It is noted that NS remission rates are considered high in our MCD, FSGS, and MN patients with 35 (84%), 40 (77%), and 21 (67%) respectively, hence it is unknown if the limited effectiveness of statin is class driven or due to resolving dyslipidemia in remitting NS.

Our study is limited by its retrospective nature and relatively small sample. In addition, the data were extracted from one center and all cohort are of Arab descent (Saudi), patients had unknown dietary habits, their baseline lipid profile before NS presentation was mostly not performed, and confirmation of compliance to statin was only possible through reviewing documents. Also, the follow up period was limited to only 12-months.

## Conclusions

It appears that primary NS might differ in initial lipid profile presentation depending on the culprit primary glomerulopathy; such a difference might be practically helpful in determining the likelihood of underlying glomerulopathy, especially in situations when kidney biopsy is risky or difficult to obtain. Moreover, this distinctive variation might open the door for a better understanding of the mechanisms resulting in NS and its related dyslipidemia. Finally, patients might be deemed to have dyslipidemia for the rest of their lives despite resolving NS, and unneeded treatment might last for several years exposing the patients for unnecessary drugs. Hence, treatment of dyslipidemia in NS might not be as necessary as in other conditions, especially when signs of NS remission are being observed early in the disease process.
